# Applications of polymeric nanoparticles in drug delivery for glioblastoma

**DOI:** 10.3389/fphar.2024.1519479

**Published:** 2025-01-06

**Authors:** Shuhan Liu, Bin Tan, Feng Wang, Ying Yu

**Affiliations:** ^1^ Department of Neurosurgery, The First Hospital, Jilin University, Changchun, Jilin, China; ^2^ Key Laboratory of Organ Regeneration and Transplantation of Ministry of Education, Institute of Immunology, The First Hospital, Jilin University, Changchun, Jilin, China; ^3^ Cancer Center, The First Hospital, Jilin University, Changchun, Jilin, China; ^4^ National-Local Joint Engineering Laboratory of Animal Models for Human Diseases, Changchun, Jilin, China

**Keywords:** glioblastoma, nanoparticles, polymer, drug delivery, treatment

## Abstract

Glioblastoma (GBM) remains one of the most aggressive and treatment-resistant brain tumors, necessitating innovative therapeutic approaches. Polymer-based nanotechnology has emerged as a promising solution, offering precise drug delivery, enhanced blood-brain barrier (BBB) penetration, and adaptability to the tumor microenvironment (TME). This review explores the diverse applications of polymeric nanoparticles (NPs) in GBM treatment, including delivery of chemotherapeutics, targeted therapeutics, immunotherapeutics, and other agents for radiosensitization and photodynamic therapy. Recent advances in targeted delivery and multifunctional polymer highlight their potential to overcome the challenges that GBM brought, such as heterogeneity of the tumor, BBB limitation, immunosuppressive TME, and consideration of biocompatibility and safety. Meanwhile, the future directions to address these challenges are also proposed. By addressing these obstacles, polymer-based nanotechnology represents a transformative strategy for improving GBM treatment outcomes, paving the way for more effective and patient-specific therapies.

## 1 Introduction

Glioblastoma (GBM) is the most common and aggressive primary malignant brain tumor in the central nervous system (CNS), known for its complex treatment challenges and poor prognosis. Characterized by marked cellular heterogeneity, rapid proliferation, invasive growth, extensive angiogenesis, and necrosis (Zlokovic, 2008), GBM originates from malignant astrocytic transformation, exhibiting high cellular polymorphism, elevated mitotic rates, and significant microvascular proliferation. The World Health Organization (WHO) classifies GBM as a grade IV glioma, the most malignant category. Based on molecular profiling, GBM is further categorized into subtypes—classical, mesenchymal, neural, and proneural—each with distinct molecular and clinical features (Murphy and Rabkin, 2013). Epidemiologically, GBM accounts for roughly 15% of all brain and CNS tumors, with an incidence rate of 3.2 per 100,000 people, primarily affecting adults aged 45–70 (Ling et al., 2023). Although rare, GBM can also occur in children and adolescents, with a slightly higher prevalence in males. Despite current standard treatments, including surgery, radiotherapy, and chemotherapy, prognosis remains poor: median survival is limited to 14–16 months, and the 5-year survival rate is less than 10%. Prognostic factors include patient age, tumor size and location, extent of surgical resection, and tumor-specific genetic markers. For instance, methylation of the MGMT (O6-methylguanine-DNA methyltransferase) gene promoter is a strong predictor of response to temozolomide (TMZ) therapy (Kumari et al., 2023). However, even with optimized treatment, long-term survival in GBM patients remains extremely low (Huang et al., 2024).

**FIGURE 1:**
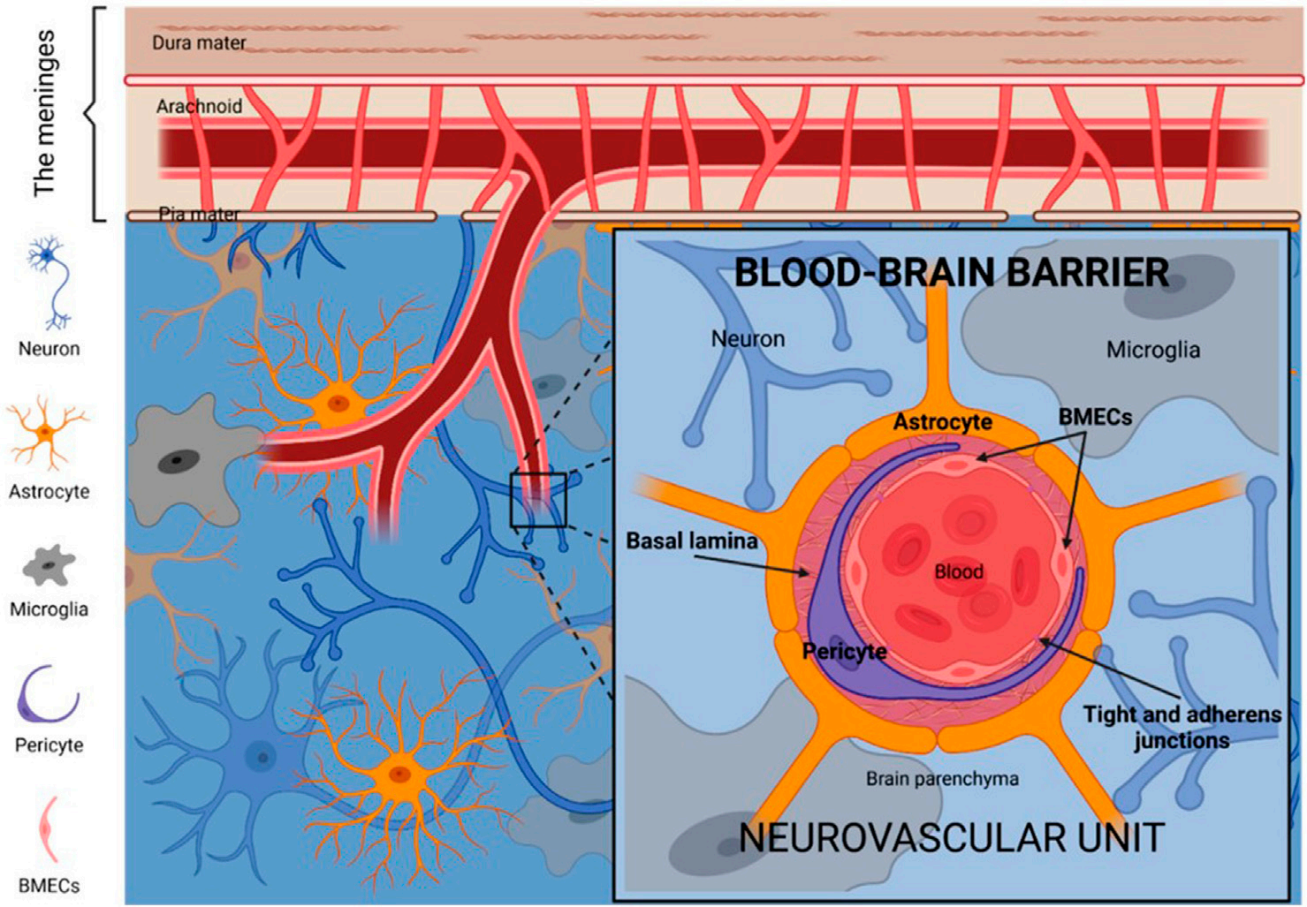
Illustration of the basic structure and components of the BBB (Ruiz-López and Schuhmacher, 2021).

The treatment of GBM is fraught with significant challenges, primarily stemming from its aggressive invasiveness and the protective role of the blood-brain barrier (BBB) (Figure 1) (Zlokovic, 2008). GBM cells readily infiltrate adjacent brain tissue, making complete surgical resection nearly unachievable. Despite advancements in microsurgical and imaging techniques, recurrence remains a frequent outcome. Furthermore, the high degree of heterogeneity within GBM cells complicates treatment, as variations in cellular structure and molecular characteristics lead to inconsistent therapeutic responses and increased drug resistance (Le Rhun et al., 2019). As a result, conventional approaches—including surgery, radiotherapy, and chemotherapy—often yield limited efficacy. The BBB compounds these challenges by restricting the delivery of therapeutic agents to the brain (Le Rhun et al., 2019). Comprised of tightly joined endothelial cells, the BBB serves a protective role but simultaneously impedes the passage of chemotherapeutic agents and emerging therapies, such as immunotherapy and targeted treatments (Liu et al., 2022a). To overcome this barrier, researchers have explored methods such as hyperosmotic agents, ultrasound-assisted drug delivery, and nanoparticle (NP)-based design. While these techniques can enhance drug penetration to a degree, clinical implementation remains challenging (Xiao et al., 2022). In summary, the invasive properties of GBM and the restrictive nature of the BBB substantially limit treatment outcomes. Although current therapies modestly extend survival, high recurrence rates and limited long-term survival highlight the urgent need for innovative approaches.

Nanotechnology presents significant potential for overcoming the challenges of GBM treatment, particularly in circumventing the BBB and enabling targeted drug delivery (Kane et al., 2015). NPs have demonstrated the capacity to cross the BBB due to their small size and customizable surface properties, allowing for enhanced penetration and accumulation within the brain (Xiao et al., 2022). Polymeric NPs, specifically, offer unique benefits in GBM treatment. These particles exhibit excellent biocompatibility, reducing the likelihood of adverse immune responses, and their degradation rates can be engineered by selecting or modifying specific polymers, such as poly(lactic-co-glycolic acid) (PLGA) or polylactic acid (PLA). This adjustability allows for controlled drug release tailored to the GBM tumor environment, maintaining therapeutic levels over extended periods. Furthermore, the versatility of polymeric NPs supports multifunctional designs, enabling simultaneous drug delivery, imaging, and potentially synergistic therapies. Together, these properties highlight the promise of polymer-based nanotechnology in advancing GBM treatment.

The primary aim of this review is to summarize and evaluate recent advancements in the application of polymeric NPs for drug delivery in GBM treatment. By examining various types of polymeric NPs, targeted delivery strategies, and multifunctional approaches, this review highlights how these innovations address critical challenges posed by GBM, such as the BBB and tumor heterogeneity. Additionally, this review aims to identify future directions for polymeric NP-based therapies, exploring emerging strategies to enhance delivery efficiency, specificity, and safety, thus offering insights into potential avenues for clinical translation in GBM treatment.

## 2 Polymeric NPs drug delivery system

Polymeric NPs represent a versatile and promising drug delivery platform, particularly for challenging applications such as GBM treatment. These nanoscale carriers are formed from biodegradable polymers that allow for controlled and sustained release of therapeutic agents, which is essential in maintaining effective drug concentrations within the tumor over time (Miranda et al., 2017). By enhancing drug stability and reducing degradation, polymeric NPs significantly improve bioavailability (Abbasi et al., 2024; Beirampour et al., 2024). Furthermore, surface modifications with targeting ligands—such as antibodies, peptides, or small molecules—allow polymeric NPs to bind specifically to tumor cells, thereby enhancing drug accumulation in tumors while minimizing toxicity to healthy tissues (Murphy and Rabkin, 2013). Physical and chemical modifications also enhance polymeric NPs’ ability to penetrate the BBB, increasing drug distribution and concentration in brain tissues (Kumari et al., 2023). Due to their excellent biocompatibility and tunable properties, polymeric NPs have been explored widely in GBM treatment.

### 2.1 Types of polymeric NPs

**FIGURE 2:**
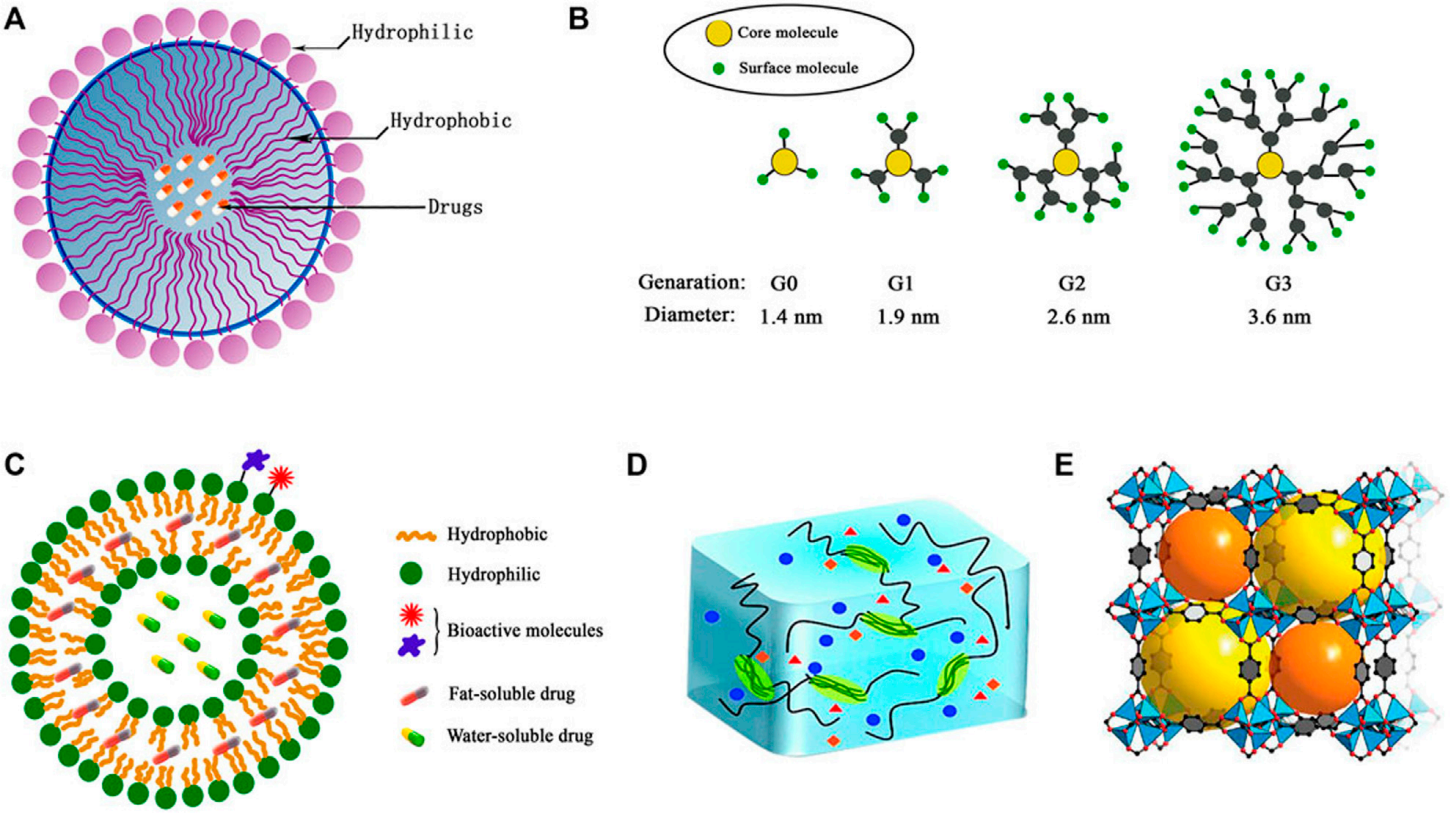
Structures of typical polymeric NPs **(A)** micelles, **(B)** dendrimers, **(C)** polymersomes, **(D)** hydrogel, **(E)** metal–organic framework (MOF) (Xiao et al., 2022).

Diverse range of polymeric NPs have been studied in drug delivery systems for GBM treatment. These NPs vary in structure, composition, and functional capabilities, each offering unique benefits for targeted therapeutic applications (Table 1). Common types include micelles, dendritic polymers, polymer vesicles, hydrogels, and metal-organic frameworks (MOFs) (Figure 2).

**TABLE 1:** Comparison of different types of polymeric NPs.

Type	Structure	Key characteristics	Advantages	Limitations
Micelles	Spherical, with a hydrophobic core and hydrophilic shell	Self-assembled from amphiphilic polymers; capable of encapsulating hydrophobic drugs	High drug-loading capacity for hydrophobic drugs; controlled drug release; biocompatible	Limited stability *in vivo*; potential premature drug release
Dendritic polymers	Highly branched, tree-like structures	Monodisperse, precise molecular weight, functionalizable surface	Precise drug loading; high surface area for functionalization; effective in delivering nucleic acids or small molecules	High production cost; potential cytotoxicity from unmodified structures
Polymer vesicles	Spherical bilayer structures resembling liposomes	Composed of amphiphilic polymers forming a bilayer; encapsulate both hydrophilic and hydrophobic drugs	Can deliver both water- and oil-soluble drugs; tunable bilayer thickness and stability	Fragile under shear stress; possible leakage of drugs
Hydrogels	3D polymeric networks with high water content	Hydrophilic, biocompatible, and capable of encapsulating large molecules	Excellent biocompatibility; ability to release drugs in response to environmental stimuli	Poor mechanical strength; potential for burst release
MOFs	Porous, crystalline structures formed from metal ions and organic linkers	High surface area, tunable porosity, and chemical stability	Large drug loading capacity; ability to protect sensitive drugs (e.g., proteins, siRNA); stimuli-responsive release	Metal toxicity concerns; complex synthesis

#### 2.1.1 Micelles

Micelles are self-assembled structures characterized by a hydrophobic core and hydrophilic shell, which impart amphiphilic properties (Kreuter, 2001). These properties enable micelles to encapsulate hydrophobic drugs and release them under specific conditions, with controlled release influenced by factors such as pH, temperature, or enzymatic activity (Xiao et al., 2022). Such controlled release enhances drug efficacy at the target site. Additionally, micelles offer distinct advantages, including improved solubility and targeted delivery for hydrophobic drugs (Ferraro et al., 2024). Studies indicate that polymeric micelles facilitate effective drug release and cellular uptake, thus enhancing therapeutic outcomes (Mora-Cabello et al., 2024). Their high biocompatibility further allows stable *in vivo* presence, minimizing toxic side effects.

Micelle properties—such as size, shape, and surface characteristics—can be tailored by selecting different polymers and synthesis conditions to suit various applications (Shishlyannikov et al., 2024). Beyond drug delivery, micelles can also be functionalized to carry imaging agents or diagnostic reagents, broadening their applications in fields such as cosmetics, biomedical imaging, and targeted drug delivery (Sun et al., 2008). pH-sensitive micelles, for instance, are amphiphilic polymers that undergo structural changes in response to pH fluctuations (Ali et al., 2021). In different pH environments, ionic groups on the polymer chains become protonated or deprotonated, causing the micelles to disassemble or re-aggregate, thereby controlling drug release (Almoustafa et al., 2021). This mechanism enables drug release in specific pathological environments, such as tumor microenvironments (TMEs), enhancing therapeutic effects while minimizing harm to healthy cells.

Reduction-responsive micelles are nanoscale structures composed of polymer surfactants, designed to release drugs or active ingredients through reduction reactions under specific conditions (Dugas et al., 2019). In the presence of reductants, these micelles undergo structural changes that facilitate controlled release (Franco et al., 2023). By concentrating drugs at the target site, they enhance therapeutic effects while minimizing side effects and limiting damage to healthy cells (Gad et al., 2016). With their high biocompatibility and tunable release properties, reduction-responsive micelles offer an effective system for targeted drug delivery.

Photosensitive micelles are formed by the self-assembly of zwitterionic polymers that respond to specific wavelengths of light (Geszke-Moritz and Moritz, 2024). Composed of both hydrophilic and hydrophobic segments, these micelles encapsulate drugs and release them upon light exposure. Photosensitizers within the micelles initiate photochemical reactions, leading to either drug release or micelle depolymerization (Heon Lee et al., 2019). Light intensity and wavelength can be adjusted to control release rates precisely, minimizing potential impact on healthy tissues (Hickey et al., 2015). Furthermore, when combined with imaging techniques, photosensitive micelles facilitate real-time monitoring of drug release and therapeutic efficacy.

#### 2.1.2 Dendritic polymers

Dendritic polymers are highly branched structures synthesized via polymerization techniques and are typically classified into dendrimers and dendritic polymer networks (Ho et al., 2012). These polymers, characterized by repetitive branching units, create highly symmetrical and versatile platforms with wide-ranging applications in drug delivery, gene transfer, imaging, and sensing (Xiao et al., 2022). As drug carriers, dendritic polymers deliver therapeutics directly to target cells or tissues, enhancing efficacy while minimizing side effects. Their surfaces can be modified with functional groups for targeted delivery, and their small size and surface characteristics facilitate efficient cellular uptake through endocytosis (Karim et al., 2016). Furthermore, their porous structure allows controlled drug release under specific conditions, such as pH fluctuations or enzymatic presence, which contributes to effective therapeutic outcomes (Kauser et al., 2023). Research has demonstrated that the tunable structure and biocompatibility of dendritic polymers improve drug bioavailability and therapeutic effects, with potential for enhanced DNA transfection efficiency in gene transfer (Kim et al., 2021).

Divergent dendritic polymers are synthesized in a stepwise manner, resulting in precise molecular structures with multiple functional sites (Koti et al., 2024). Their highly branched architecture provides a large surface area for binding drugs or genes, forming stable carriers that improve solubility and bioavailability while preserving therapeutic activity *in vivo* (Li et al., 2017a). These polymers can also integrate multiple therapeutic or imaging functionalities within a single carrier, enhancing targeting capabilities.

Convergent dendritic polymers, constructed via convergent synthesis methods, also feature multi-branched structures (Liu et al., 2018). Compared to linear or crosslinked polymers, these dendritic polymers exhibit unique spatial configurations and functional versatility (Marshall et al., 2022). Their surfaces can be modified with targeting groups to facilitate binding with specific cells or tissues, allowing sustained release triggered by environmental factors (e.g., pH or temperature changes). This dendritic structure promotes cell membrane penetration and enhances drug endocytosis (Muhtadi et al., 2020). Through surface modifications, convergent dendritic polymers support diverse functionalities, improving drug delivery efficiency and bioavailability beyond traditional carriers (Nance et al., 2014).

#### 2.1.3 Polymer vesicles

Polymer vesicles are self-assembled structures formed from amphiphilic polymers, with a structure resembling that of liposomes (Naser et al., 2024). Their formation involves selecting suitable polymers and employing methods such as solvent evaporation or solution self-assembly to produce stable vesicles (Xiao et al., 2022). This structural stability helps maintain drug efficacy *in vivo* by protecting against degradation (Niza et al., 2021). Polymer vesicles can enter cells through endocytosis to release their contents intracellularly. Additionally, surface modifications enable targeted delivery to specific cells or tissues, enhancing therapeutic efficacy (Nozohouri et al., 2019). Beyond drug delivery, polymer vesicles serve as gene carriers for DNA or RNA and as vaccine carriers to boost immune responses.

Thermo-sensitive polymer vesicles are polymer particles that respond to temperature changes by altering their physical or chemical properties (Ou et al., 2024). Typically, their mechanism involves conformational shifts in the polymer chains at a critical temperature, affecting solubility and biomolecular interactions (Pillai et al., 2020). For instance, poly(N-isopropylacrylamide) is a commonly used thermo-sensitive polymer that transitions from hydrophilic to hydrophobic above its critical temperature, enabling controlled drug release (Raman et al., 2020). Drugs can be loaded at temperatures below this threshold, and upon reaching the critical temperature, the polymer collapses, releasing the drugs (Ramírez-García et al., 2019). This rapid thermal response allows for precise control over drug release rates, enhancing therapeutic effects and reducing side effects in non-target tissues (Sabit et al., 2022). Thermo-sensitive polymers are also employed in bioimaging and sensor development.

pH-sensitive polymer vesicles are carriers that alter their physical or chemical properties in response to environmental pH changes (Ramírez-García et al., 2019). Composed of polymers with acidic or basic groups, these vesicles exhibit reversible solubility or aggregation under specific pH conditions, facilitating controlled drug release (Cheng et al., 2019). At designated pH levels, their solubility changes, triggering release in targeted environments, such as the acidic TME (Sartaj et al., 2021). pH-sensitive vesicles are also valuable in bioimaging as contrast agents and in biosensors for biomarker detection (Song et al., 2024). Additionally, they can be combined with other functionalities, such as temperature or light sensitivity, to enable more complex therapeutic applications.

#### 2.1.4 Hydrogels

Hydrogels consist mainly of hydrophilic polymers that form three-dimensional networks in water, endowing them with excellent biocompatibility (Qin et al., 2014), flexibility, and moisture retention properties. Their structural stability arises from mechanisms such as physical adsorption, chemical bonding, and reversible expansion or contraction (Wu et al., 2021). These features make hydrogels highly suitable for various biomedical applications, including drug delivery, tissue engineering, and biosensing (Xu et al., 2023). In drug delivery, hydrogels encapsulate drugs efficiently and control their release, thereby enhancing bioavailability (Yang et al., 2024). As scaffolds, they support cell growth and tissue regeneration, while their high hydration capacity and conductivity make them ideal for biosensor development (Zhang and Tung, 2017).

Temperature-sensitive hydrogels are polymer networks that change their hydration state and structure within specific temperature ranges (typically near the critical dissolution temperature), allowing them to absorb or release water as needed (Zubris et al., 2012). As temperature increases, these hydrogels undergo phase transitions or chemical interactions that compact the polymer chains, leading to water expulsion and volume reduction (Zu et al., 2021). Polymer composition and crosslinking density can be adjusted to tune their properties. Temperature-sensitive hydrogels are biocompatible and non-toxic, commonly used to regulate drug release rates and facilitate drug delivery at targeted temperatures (Zhang et al., 2018). They are also applied in tissue engineering and biosensing.

pH-sensitive hydrogels respond to environmental pH changes by altering their physical and chemical properties (Ramírez-García et al., 2019). The functional groups on the polymer chains (e.g., acidic or basic groups) undergo ionization or deionization at different pH levels, causing changes in hydrophilicity and swelling, which in turn modulates the release rates of encapsulated substances (Xu et al., 2023). Their responsiveness can be fine-tuned by modifying polymer chemical structures and compositions. pH-sensitive hydrogels enable controlled drug release in specific pH environments (such as TMEs or the gastrointestinal tract) and are widely used in tissue engineering (Zhang et al., 2021), biosensors, and environmental protection due to their biocompatibility and adjustable release characteristics.

#### 2.1.5 Metal-organic frameworks (MOFs)

Metal-organic frameworks (MOFs) are porous materials created through the coordination of metal ions or clusters with organic ligands (Amin et al., 2024), yielding high surface areas and adjustable pore sizes suited for diverse applications. The porous structure and surface functional groups of MOFs facilitate interactions with drug molecules, enabling efficient drug adsorption, release, and targeted delivery—properties valuable in drug delivery systems (Gandhi et al., 2024). Their biocompatibility and low toxicity further enhance their potential for medical applications (Harwansh et al., 2024).

MOFs are highly ordered and customizable, with tunable pore sizes and chemical environments that can be optimized by selecting specific metals and ligands for various uses (Choi et al., 2020). Their high surface areas provide numerous active sites, increasing adsorption capacity and enhancing drug loading and release efficiency (Silva et al., 2024). Beyond drug delivery, MOFs are employed in gas capture (e.g., carbon dioxide and hydrogen storage) and as catalysts or catalyst supports, improving reaction selectivity and activity (Gawel et al., 2024). Additionally, most MOFs exhibit stable physicochemical properties under humid or high-temperature conditions and can be synthesized from renewable materials (Bhanja et al., 2023), offering environmentally friendly advantages.

Stimuli-responsive MOFs, assembled from metal ions and organic ligands, offer tunable porosity and can adjust their properties in response to external stimuli (Caverzan and Ibarra, 2024). For example, changes in pH can protonate organic ligands, modifying pore size and influencing drug release rates. In biomedical applications, these MOFs enable targeted, controlled drug delivery, reducing side effects (Chang et al., 2024). Furthermore, they are advantageous for gas storage and separation, as well as in sensing and catalysis, with specific functionalities achievable through simple chemical modifications.

### 2.2 Targeted delivery strategies and multifunctionality of polymeric NPs

Targeted delivery strategies using polymeric nanodrugs in GBM treatment provide innovative approaches to enhance therapeutic efficacy (Ullah et al., 2024). Through ligand modification and environment-responsive designs, these strategies allow for precise tumor targeting and localized drug release (Chang et al., 2024). Multifunctional nanodrugs, particularly those that integrate chemotherapy, radiotherapy, and immunotherapy, have shown further improvements in treatment outcomes (Kreuter, 2001).

#### 2.2.1 Targeted nanodrug design

The targeted delivery of polymeric nanodrugs involves designing nanocarriers that selectively target tumor cells while minimizing effects on normal cells. Key strategies include targeted ligand modification and environment-responsive designs. Targeted ligand modification decorates nanodrug surfaces with specific ligands that bind to receptors overexpressed on tumor cells, such as transferrin receptor (TfR), folate receptor (FR), and epidermal growth factor receptor (EGFR) (Dhar et al., 2022). For instance, polymeric NPs modified with EGF can target GBM cells overexpressing EGFR, while VEGF-modified polymeric NPs can enhance targeting of the TME, specifically within the tumor vasculature (Khan et al., 2022). Moreover, antibody modification is another common method for enhancing polymeric NP targeting specificity. Antibodies provide high specificity by recognizing unique antigens on tumor cells, directing NPs precisely to tumor sites (Kreatsoulas et al., 2022). For example, anti-PD-L1 antibodies conjugated to NPs can selectively target cells within the TME that express PD-L1, which is beneficial for improving the specificity of immunotherapy (Lee et al., 2022). Modifications using aptamers and peptides also enhance targeting specificity (Dhayalan et al., 2024). Aptamers and short peptides are small-molecule targeting ligands with high specificity, cost-efficiency, and stability. Aptamers are specific DNA or RNA sequences, while peptides are composed of specific amino acid sequences, both of which can recognize and bind to unique tumor cell markers (Morales and Mousa, 2022). RGD peptides, for example, can bind to integrin receptors on tumor-associated blood vessels in GBM, facilitate GBM targeting (Kreatsoulas et al., 2022). Furthermore, multivalent ligand modification, involving multiple ligands on a single nanodrug, improves binding affinity and drug accumulation at tumor sites through interactions with multiple receptors. In short, these diverse targeting strategies modify the NP surface with specific ligands to enhance accumulation at tumor sites and improve therapeutic efficacy.

Environment-responsive nanodrugs are designed to release drugs in response to conditions within the TME, including pH, enzyme presence, temperature, or oxygen levels (Zhang et al., 2024b). This approach takes advantage of the unique properties of tumor sites to achieve selective drug release and enhance targeted drug delivery (Mu et al., 2024). For instance, TMEs often exhibit slightly acidic pH values (around 6.5–6.8) compared to normal tissues. pH-responsive polymeric NPs are designed to release their payload when exposed to these acidic conditions, ensuring drug release occurs primarily within the tumor site (Chen et al., 2020). These NPs can be engineered with acid-sensitive linkages or polymers that undergo structural changes in acidic conditions, and polymers containing groups like poly(β-amino esters) or imidazole undergo protonation in acidic environments, leading to destabilization and drug release. What’s more, many tumors overexpress specific enzymes, such as matrix metalloproteinases (MMPs), cathepsins, and hyaluronidases, which play roles in tumor invasion and metastasis. Enzyme-responsive NPs are engineered to degrade or release drugs in response to these enzymes, targeting the TME specifically (Gallego and Ceña, 2020). For instance, polymeric NPs containing MMP-sensitive linkers have been developed for selective release in tumor tissue, while hyaluronic acid-based polymeric NPs degrade in the presence of hyaluronidase, releasing the drug near cancer cells overexpressing this enzyme (Senobari et al., 2024). In addition, Temperature-responsive nanodrugs are designed to release drugs in response to elevated temperatures, a property that can be exploited by externally applied heat or hyperthermia in cancer treatment. Temperature-sensitive polymers, such as poly(N-isopropylacrylamide) (PNIPAM), undergo a phase transition at specific temperatures, releasing the drug in response to hyperthermic conditions (Shaibie et al., 2023). Lastly, oxygen-responsive nanodrugs release drugs under hypoxic conditions typical of TMEs, often utilizing redox-sensitive groups for controlled release. These NPs are engineered with disulfide linkages or redox-sensitive polymers that cleave in the presence of high GSH levels, leading to controlled drug release inside tumor cells. Once the NPs are internalized by tumor cells, the high GSH concentration triggers the breakdown of disulfide bonds, releasing the drug specifically in the tumor cells (Li et al., 2017b). For example, redox-responsive micelles or nanovehicles with disulfide linkages in their core have shown efficient intracellular drug release in cancer cells with elevated GSH levels, while remaining stable in the bloodstream.

#### 2.2.2 Multifunctionality of polymeric NPs

Multifunctional nanodrugs integrate therapeutic agents and functional modules to enable multi-targeted combination therapy, providing comprehensive effects against tumors (Ghasempour et al., 2022). These nanodrugs co-deliver multiple therapeutics such as chemotherapeutics, radiotherapy sensitizers, and immune modulators to improve GBM treatment outcomes. Moreover, they can also be engineered to function as theranostic agents, combining therapeutic and diagnostic functions within the same particle.

Polymeric NPs are well-suited for the co-delivery of multiple therapeutic agents within a single carrier. This strategy allows for synergistic effects, where drugs with complementary mechanisms can enhance overall efficacy. The combination of chemotherapy and radiotherapy involves loading both chemotherapeutic drugs and radiotherapy sensitizers into nanodrugs to enhance tumor cell sensitivity and boost therapeutic efficacy. For instance, nanodrugs encapsulate chemotherapeutics such as doxorubicin, cisplatin, or TMZ to improve drug stability and bioavailability (Kang et al., 2018). Radiotherapy sensitizers like gold NPs or cisplatin can also be incorporated to increase tumor radiosensitivity. For example, gold-based NPs enhance radiation absorption due to their high atomic number, thereby amplifying radiation-induced damage to tumor cells (Karlsson et al., 2021). Another application involves the co-delivery of immunotherapy agents, such as PD-L1 inhibitors, along with chemotherapeutics, aiming to enhance immune response against GBM cells. Beyond the above therapeutics, polymeric NPs are capable of delivering gene therapy agents and anti-angiogenic drugs (Formica et al., 2021). Gene therapy NPs carrying siRNA or antisense oligonucleotides enhance transfection efficiency in tumor cells, enabling selective oncogene silencing (Lansangan et al., 2024). Moreover, loading anti-angiogenic drugs onto gold-PLGA composite NPs improves targeting of tumor blood vessels, inhibiting angiogenesis and reducing tumor blood supply.

In addition to co-delivery of multiple therapeutics, polymeric NPs can also be engineered to function as theranostic agents, combining therapeutic and diagnostic functions within the same particle. This dual functionality enables simultaneous treatment and real-time monitoring of therapeutic outcomes, which is particularly useful in aggressive cancers like GBM (Morales and Mousa, 2022). Certain NPs can be co-loaded with drugs and imaging agents, such as MRI contrast materials or fluorescent markers, allowing for the visualization of drug distribution and tumor response (Khan et al., 2022).

Overall, polymeric NPs offer a highly adaptable and multifunctional platform for GBM drug delivery. By exploring various polymeric NP types—including micelles, dendritic polymers, vesicles, and hydrogels—each structure provides distinct advantages for encapsulating and delivering therapeutics. Targeted delivery strategies, through ligand modification and environment-responsive designs, further enhance specificity, allowing NPs to precisely release drugs within the TME. Additionally, multifunctional NPs facilitate the co-delivery of therapeutics, achieving synergistic effects that improve therapeutic efficacy. Moreover, theranostic applications of NPs enable real-time monitoring of treatment and disease progression. Together, these attributes make polymeric NPs a promising avenue for advancing targeted, controlled, and effective GBM treatments, addressing the complex challenges posed by tumor heterogeneity and the BBB.

## 3 Application of polymeric NPs in GBM treatment

Traditional treatments for GBM, including surgery, radiotherapy, and chemotherapy, have shown limited efficacy (Lim et al., 2024). The development of polymeric NPs offers new possibilities for GBM therapy by providing multifunctional NPs with strong biocompatibility, efficient drug delivery, and targeting abilities (Lim et al., 2023). These NPs enable precise drug delivery, targeted therapy, and combination therapies by delivering chemotherapeutics, targeted therapeutics, immunotherapeutics, and other agents, addressing the limitations of conventional treatments (Table 2).

**TABLE 2:** Examples of NP delivery for different types of therapeutic drugs.

Treatment type	Payload	NP type	Advantages	Ref
Chemotherapy	TMZ	PEI NPs	Improved drug delivery efficiency across the BBB and targeted glioma cells	Lin et al. (2024a)
Chemotherapy	Doxorubicin	PEG-SS-PLA	Enhanced GBM chemotherapy by overcoming drug resistance and the BBB	Wang et al. (2023a)
Targeted Therapy	Cas9 protein, sgRNA targeting STAT3	Lipid-polymer hybrid NPs	Achieved vasculature normalization and immune reprogramming for GBM treatment	Zhang et al. (2024a)
Targeted Therapy	VEGF N-terminal epitope	Molecularly imprinted polymer NPs	Accumulated in tumor sites due to the elevated VEGF levels in the TME	Zhao et al. (2023)
Immunotherapy	PD-L1 siRNA	Semiconducting polymer	Leveraged neutrophils’ natural migration across the BBB to achieve targeted delivery to glioma sites	Ding et al. (2024)
Immunotherapy	CD47 and PD-L1 siRNA	Lipid NPs	Improved transfection efficiency, specific gene silencing	Liu et al. (2022a)
Other Therapy	Radiotherapy Sensitizers	Iron Oxide NPs	Enhanced radiotherapy sensitivity	Tang et al. (2023)
Other Therapy	Anti-Angiogenic Drugs	Gold-PLGA Composite NPs	Enhanced targeting of tumor blood vessels	Zhang et al. (2022)

### 3.1 Polymeric NPs deliver chemotherapeutic agents

Chemotherapy is a cornerstone in GBM treatment, with TMZ being the most widely used agent. However, its efficacy is limited by challenges such as the BBB, systemic toxicity, and acquired drug resistance. These limitations reduce the therapeutic index, making novel delivery systems essential. Polymeric NPs offer a promising approach by improving drug stability, reducing off-target effects, and enhancing accumulation in the TME. Polymeric NPs provide a protective shell around chemotherapeutic drugs, shielding them from enzymatic degradation and premature clearance. Biodegradable polymers such as PLGA and PLA are commonly used to encapsulate drugs, ensuring sustained delivery and prolonged circulation time. PEGylation, or the addition of polyethylene glycol (PEG) to the NP surface, further extends systemic circulation by reducing immune clearance. These modifications improve the bioavailability of drugs like TMZ, enabling them to cross the BBB more effectively (Ramalho et al., 2023).

**FIGURE 3:**
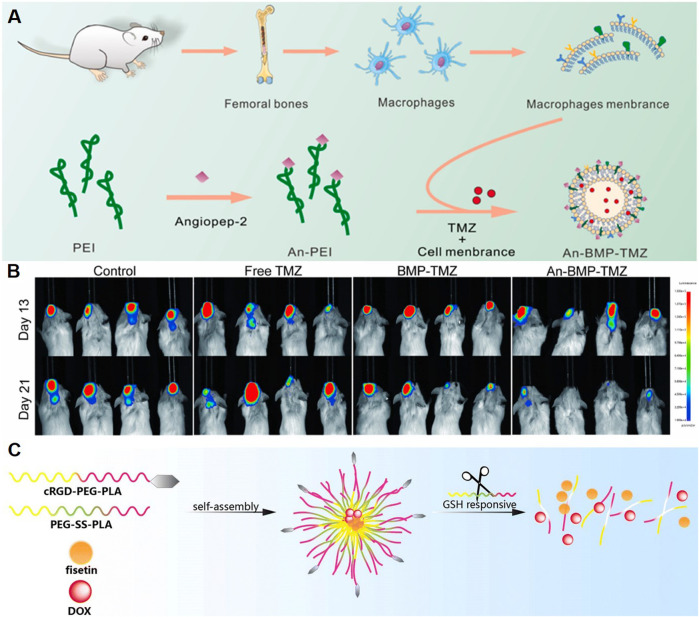
Polymeric NPs Deliver Chemotherapeutic Agents. **(A)** Schematic illustration of the design of An-BMP-TMZ. **(B)** Luminescence images of U87-Luc^+^ tumor-bearing C-NKG mice following different treatments and monitored on days 13 and 21 (Lin et al., 2024b). **(C)** Schematic illustration of the system co-loading DOX and fisetin by cRGD-decorated NPs (Wang et al., 2023a).

Recent studies highlight the potential of polymeric NPs in enhancing chemotherapeutic delivery for GBM. TMZ, a first-line chemotherapeutic drug for GBM, was loaded in NPs to improve therapeutic effect. A kind of polyethyleneimine (PEI)-based polymeric NP was reported to treat GBM by combining macrophage membrane-coated NPs with low-frequency ultrasound (LFU) irradiation (Figure 3A) (Lin et al., 2024b). The NPs, synthesized using PEI modified with angiopep-2, were coated with macrophage membranes to improve biocompatibility and immune evasion. Encapsulating TMZ, the NPs demonstrated a drug loading efficiency of 44.2%. LFU irradiation was used to temporarily open the BBB, and angiopep-2, which binds to LRP receptors, was utilized for targeting glioma cells. This system exhibited improved tumor inhibition, reduced systemic toxicity, and increased survival rates in animal models (Figure 3B).

Other chemotherapeutic agents have also been explored by polymer NPs loading for GBM treatment. Wang et al. designed a nano-drug delivery system (Fis-DOX/cRGD-NPs) to enhance GBM chemotherapy by overcoming drug resistance and the BBB (Figure 3C) (Wang et al., 2023a). The NPs were synthesized using PEG-SS-PLA and cRGD-PEG-PLA, loaded with fisetin and doxorubicin via the thin-film hydration method. The functionalization with cRGD enabled active targeting of glioma cells through integrin αvβ3 binding, while the redox-responsive disulfide bonds facilitated tumor-specific drug release in the high-glutathione environment. *In vitro* studies demonstrated enhanced cellular uptake, G2/M phase arrest, and apoptosis induction through caspase activation and BAX/BCL-2 modulation. The NPs also suppressed glioma cell proliferation, migration, and angiogenesis by inhibiting AKT and STAT3 pathways. *In vivo*, the Fis-DOX/cRGD-NPs showed superior antitumor efficacy in both subcutaneous and orthotopic glioma models, reducing tumor growth without significant toxicity. Based on these studies, polymer NPs loaded chemotherapeutic drugs show a good application prospect in the treatment of GBM.

### 3.2 Polymeric NPs deliver targeted therapeutic agents

Targeted therapy in GBM focuses on disrupting molecular pathways essential for tumor growth and survival. Polymeric NPs offer an effective strategy to deliver targeted therapeutic agents, enhancing specificity and reducing systemic toxicity. These agents often include tyrosine kinase inhibitors, monoclonal antibodies, and small molecules designed to interfere with oncogenic pathways, such as EGFR, VEGF, or PD-L1 signaling. EGFR-targeted polymeric NPs loaded with gefitinib have demonstrated increased tumor accumulation and improved therapeutic outcomes in preclinical studies (Paranthaman et al., 2020). Similarly, VEGF-targeted NPs carrying bevacizumab efficiently target tumor angiogenesis, reducing vascularization and enhancing drug delivery (Fatima et al., 2024). In addition to targeting GBM cells, polymeric NPs can address components of the TME, such as stromal cells, vasculature, and extracellular matrix. Agents like anti-angiogenic drugs delivered via NPs inhibit VEGF signaling, normalizing the tumor vasculature and improving drug penetration. And NPs encapsulating MMP-inhibiting drugs can suppress extracellular matrix remodeling, reducing tumor invasion (Islam et al., 2020).

**FIGURE 4:**
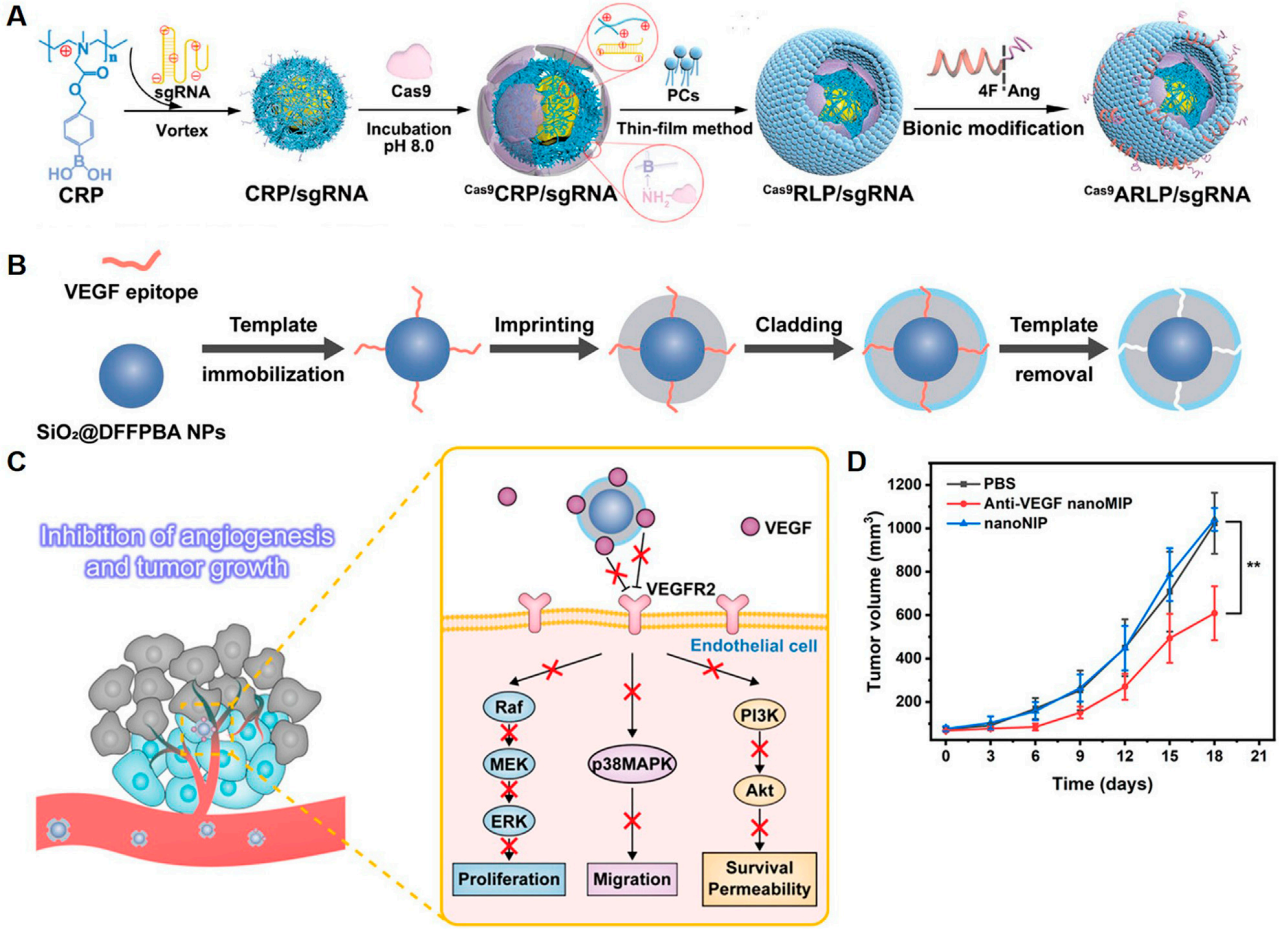
Polymeric NPs Deliver Targeted Therapeutic Agents. **(A)** Schematic illustration of Cas9ARLP/sgRNA fabrication (Zhang et al., 2024a). **(B)** Schematic of the synthesis route of anti-VEGF nanoMIP. **(C)** Schematic of using anti-VEGF nanoMIP to inhibit angiogenesis and tumor growth through binding VEGF and blocking VEGF-VEGFR2 signaling pathway. **(D)** Change of tumor volumes in different treatment groups (Zhao et al., 2023).

Some studies have explored the therapeutic efficacy of utilizing polymeric NPs to deliver drugs targeting specific sites, such as STAT3, in the treatment of GBM. Lipid-polymer hybrid NPs were reported for targeted CRISPR/Cas9-based gene editing in GBM (Figure 4A) (Zhang et al., 2024a). The NPs were designed with a cationic ROS-responsive polymer core for sgRNA condensation and Cas9 coordination, and a 4F-angiopep-2-functionalized lipid membrane to enhance BBB penetration and glioma specificity. Targeting STAT3, a key regulator of tumor angiogenesis and immunosuppression, the NPs achieved gene knockout efficiency (∼50%) in tumor models. This editing downregulated VEGF, IL-6, and IL-10 levels, resulting in tumor vasculature normalization and immune reprogramming. The system’s ROS-responsive mechanism allowed selective release of Cas9 and sgRNA in the TME. The therapy reduced tumor size and enhanced survival in orthotopic glioma models, with improved immune infiltration and activation of antitumor responses.

Polymeric NPs prepared using inorganic nanomaterials as substrates have also been explored for the treatment of GBM. A study presented a molecularly imprinted polymer NP (nanoMIP) for targeted anti-angiogenic cancer therapy, focusing on VEGF signaling (Zhao et al., 2023). The nanoMIPs were synthesized using silica-coated magnetic NPs (Fe_3_O_4_@SiO_2_) as a substrate, functionalized via boronate-affinity epitope anchoring, and imprinted with monomers to create selective binding pockets for VEGF isoforms (VEGF165 and VEGF121). Template removal ensured high-affinity, specific binding to VEGF (Figure 4B). These NPs blocked the VEGF/VEGFR2 interaction, disrupting downstream signaling pathways essential for angiogenesis (Figure 4C). *In vitro* assays demonstrated significant inhibition of endothelial cell proliferation, migration, and tube formation. *In vivo* studies showed reduced microvascular density and tumor growth suppression in xenograft models (Figure 4D). Therefore, the targeted therapeutic strategies for GBM using polymeric NPs offer a new paradigm in targeted cancer therapy.

### 3.3 Polymeric NPs deliver immunotherapeutic agents

Immunotherapy aims to harness the body’s immune system to recognize and eliminate GBM cells. Despite its success in some cancers, the highly immunosuppressive microenvironment of GBM poses significant challenges to immunotherapy. Polymeric NPs have emerged as promising tools to enhance the delivery of immunotherapeutic agents by improving their stability, targeting efficiency, and localized delivery within the TME. The TME of GBM is characterized by immune cell dysfunction, high levels of regulatory T cells (Tregs), and macrophages polarized towards an immunosuppressive M2 phenotype (Mu et al., 2024). By reprogramming the TME—such as polarizing macrophages to the M1 phenotype and suppressing Tregs—the NPs can stimulate robust antitumor immunity.

**FIGURE 5:**
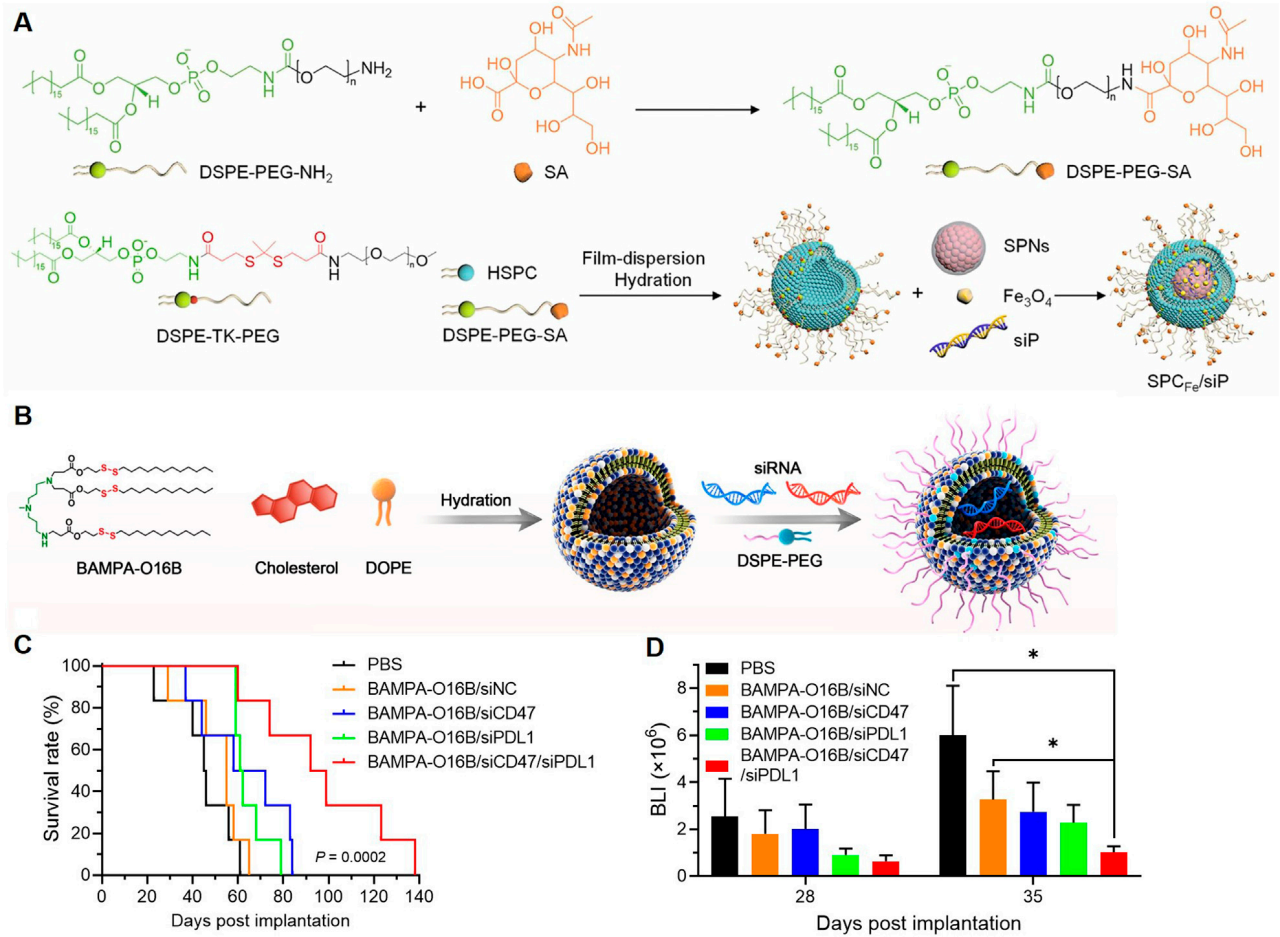
Polymeric NPs Deliver Immunotherapeutic Agents. **(A)** Diagram of the construction of SPCFe/siP (Ding et al., 2024). **(B)** Illustration of formulating bioreducible BAMPA-O16B/siRNA lipoplex. **(C)** Overall survival of GBM-bearing mice was determined by Kaplan-Meier survival analysis (*n* = 6 per group). Statistical analysis was performed using Mantel-Cox tests. **(D)** Statistical analysis of the BLI flux values of GL261-luc tumors measured at 28 and 35 days after tumor implantation (Liu et al., 2022a).

Semiconducting polymer-based materials have been reported to deliver immunotherapeutic agents. Ding et al. designed a neutrophil-based “Trojan horse” nanosystem for the treatment of GBM, combining ferroptosis and immunotherapy (Ding et al., 2024). The NPs were engineered with a semiconducting polymer, Fe_3_O_4_ NPs, and PD-L1 siRNA, encapsulated within a singlet oxygen-cleavable nanocarrier. The surface was functionalized with sialic acid-modified DSPE-PEG for neutrophil targeting (Figure 5A). The system leveraged neutrophils’ natural migration across the BBB to achieve targeted delivery to glioma sites. Upon ultrasound activation, the polymer generated singlet oxygen (^1^O₂), triggering the release of Fe_3_O_4_ and siRNA. Fe_3_O_4_ induced ferroptosis by promoting lipid peroxidation and depleting GSH, resulting in immunogenic cell death. Simultaneously, siRNA downregulated PD-L1 expression, boosting anti-tumor immune responses. The combined action of ferroptosis and immunotherapy restricted glioma growth, reduced Tregs, and prolonged survival in mouse models.

Enhancing the anti-tumor immune response by promoting the phagocytosis of macrophages has also been investigated for the treatment of GBM. A study explored the development of BAMPA-O16B lipid NPs (LNPs) for brain-targeted siRNA delivery and GBM immunotherapy (Figure 5B) (Liu et al., 2022a). The therapeutic payload included siRNAs targeting CD47 and PD-L1, two key molecules in immune suppression. *In vivo*, the dual silencing of CD47 and PD-L1 synergistically enhanced both innate and adaptive anti-tumor immunity by increasing macrophage phagocytosis, T-cell activation, and cytokine secretion. These immune modulations led to significant tumor growth inhibition and improved survival in orthotopic GBM mouse models (Figures 5C, D), which highlighted the potential of BAMPA-O16B-based LNPs for targeted CNS drug delivery and immunotherapy. In short, polymeric NPs delivering immunotherapeutic agents address critical challenges in GBM treatment, including BBB penetration, immune evasion, and effective gene silencing, paving the way for clinical translation.

### 3.4 Polymeric NPs deliver other therapeutic agents

Beyond traditional therapeutic drugs, polymer NPs can also deliver other therapeutics, such as radiotherapy sensitizers, photothermal and photodynamic agents, as well as autophagy modulation agents for GBM treatment.

**FIGURE 6:**
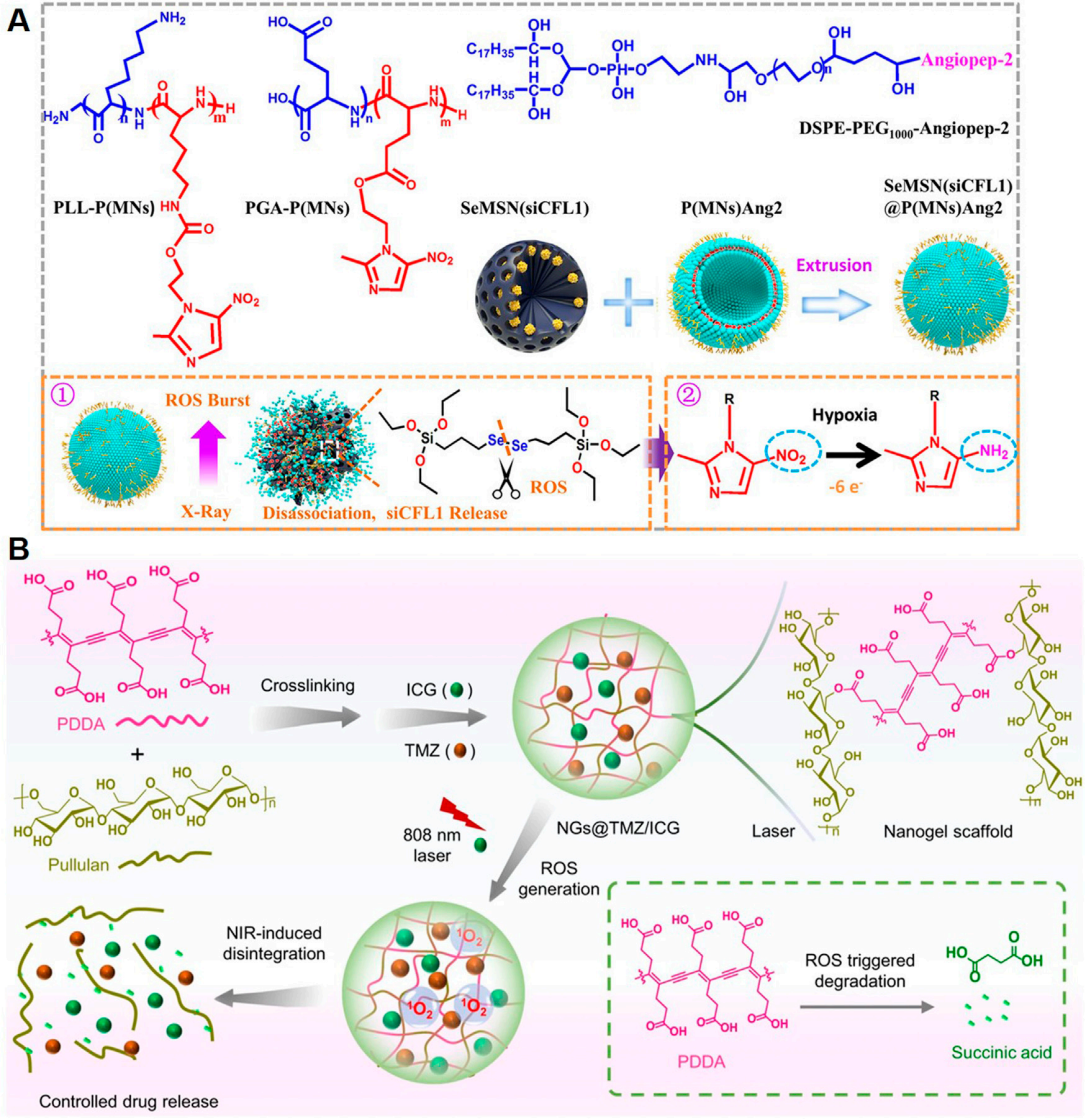
Polymeric NPs Deliver Other Therapeutic Agents. **(A)** Chemical structure of the main component of P(MNs)Ang2 and schematic showing the synthesis of SeMSN(siCFL1)@P(MNs)Ang2 (Tang et al., 2023). **(B)** Schematic illustration of the synthesis of the nanogels and their NIR-induced disintegration (Zhang et al., 2022).

Radiotherapy is a cornerstone in GBM treatment, but its efficacy is often limited by the radioresistance of tumor cells and the inability to deliver high doses without damaging healthy tissue. Polymeric NPs have emerged as effective carriers for radiosensitizing agents, enhancing the therapeutic effects of radiation. Radiosensitizers amplify DNA damage induced by radiation by increasing reactive oxygen species (ROS) production or inhibiting DNA repair mechanisms. For example, a study presented a platform utilizing selenium-engineered mesoporous silica nanocapsules (SeMSNs) for addressing radiotherapy-resistant GBM (Figure 6A) (Tang et al., 2023). The NPs were designed to deliver siRNA targeting cofilin-1 (CFL1), a key protein implicated in GBM invasion and radiation resistance. The SeMSNs were synthesized using a sol-gel process, functionalized with a hypoxia-responsive polymer coating (P(MNs)Ang2) for radiosensitization, and surface-modified with angiopep-2 for BBB penetration. Upon low-dose X-ray irradiation, ROS production triggered the disintegration of SeMSNs, releasing siRNA and radiosensitizing agents. This strategy enhanced apoptosis and inhibits GBM cell migration and invasion. The combination of RNAi and radiation sensitization achieved prolonged survival in orthotopic GBM models.

Photothermal therapy and photodynamic therapy are emerging modalities in GBM treatment, leveraging light-induced mechanisms to destroy tumor cells. Polymeric NPs are ideal carriers for photothermal agents and photosensitizers due to their stability, biocompatibility, and tunability. Polymeric NPs loaded with photothermal agents, such as polydopamine, gold NPs, or carbon dots, absorb near-infrared (NIR) light and convert it into localized heat. This selective heating induces hyperthermia, causing tumor cell death without damaging surrounding healthy tissue, therefore allowing precise targeting and controlled heat generation, which is especially beneficial in the complex brain microenvironment (Sun et al., 2022). Photodynamic therapy uses light-activated photosensitizers to generate ROS, leading to oxidative damage and cell death in tumor cells. Polymeric NPs can encapsulate photosensitizers such as porphyrins or Ce6, stabilizing them in circulation and improving tumor-specific delivery. For instance, Zhang et al. designed a nanogel system constructed from pullulan and PDDA, and loaded with the chemotherapeutic agent TMZ and the photosensitizer ICG for GBM treatment (Figure 6B) (Zhang et al., 2022). ApoE peptide-functionalized erythrocyte membranes were used to camouflage the nanogels, enabling prolonged blood circulation and active tumor targeting. NIR irradiation was applied after the nanogels accumulate in tumor lesions. The activation of ICG produced ROS, leading to nanogel deformation and controlled release of both therapeutic agents. This strategy achieved deep tumor penetration and synergized photodynamic and chemotherapeutic effects.

Autophagy, a cellular process for degrading and recycling cellular components, plays a dual role in GBM. It can either promote tumor cell survival under stress or trigger cell death when excessively activated. Polymeric NPs provide a platform for modulating autophagy to enhance GBM therapy. By delivering autophagy inhibitors, such as chloroquine or 3-methyladenine, the NPs block autophagic flux, sensitizing tumor cells to chemotherapy and radiotherapy. By preventing the protective effects of autophagy, these inhibitors enhance the cytotoxicity of standard treatments (Walweel and Aydin, 2024). In contrast, excessive autophagy can lead to programmed cell death in GBM cells. Polymeric NPs loaded with agents such as rapamycin or ceramide induce autophagy, disrupting tumor growth and progression (Agarwal and Maekawa, 2020).

In all, polymeric NPs offer innovative strategies for delivering therapeutic agents to treat GBM, addressing limitations of traditional therapies. These multifunctional systems address the complex challenges of GBM, providing targeted, controlled, and synergistic therapeutic effects while minimizing systemic toxicity, thus representing a promising direction for personalized and effective cancer treatment.

## 4 Challenges and future directions of polymer-based nanotechnology in GBM treatment

Due to limited treatment options, GBM remains one of the most difficult malignant tumors to treat clinically. While polymer-based nanotechnology offers transformative potential in overcoming the barriers of GBM, several challenges persist, including the heterogeneity of the tumor, BBB, TME, biocompatibility and safety consideration of polymeric NPs.

### 4.1 Heterogeneity of GBM

GBM is characterized by extreme inter- and intra-tumoral heterogeneity, including variations in cellular composition, genetic mutations, and signaling pathways. This complexity poses significant challenges for designing polymeric NPs that can effectively target all tumor cells. Certain GBM cells, such as GBM stem-like cells (GSCs), exhibit resistance to conventional therapies and are challenging to target with NPs. Moreover, differences in the TME across patients further complicate the design of universal therapeutic strategies (Lauko et al., 2022). Therefore, the heterogeneity of GBM presents significant challenges for polymer-based nanotechnology, particularly in achieving uniform targeting and efficacy.

Innovative future directions about GBM heterogeneity include personalization, adaptability, and combination approaches to overcome these barriers. Personalized nanomedicine is at the forefront, leveraging multi-omics data (genomics, transcriptomics, and proteomics) to design tailored polymeric NPs targeting patient-specific tumor profiles (Roque et al., 2023). Multifunctional NPs capable of co-delivering chemotherapy, gene therapy, and immunotherapy offer potential for addressing diverse tumor subpopulations. Furthermore, adaptive and stimuli-responsive NPs are being developed to dynamically respond to the TME (e.g., hypoxia, pH, and enzymatic activity) for precise and localized drug release (Jena et al., 2020). To overcome the challenge of GSCs, polymeric NPs functionalized with ligands targeting markers such as CD44 and CD133 are under investigation. Enhanced delivery systems incorporating BBB-targeting ligands like angiopep-2, along with penetration-enhancing strategies, aim to improve uniform drug distribution across heterogeneous tumor regions. Additionally, advanced preclinical models, including patient-derived xenografts and organoids, are being developed to better simulate GBM heterogeneity, enabling more predictive testing and optimization of polymer-based therapies (Jacob et al., 2020). These strategies collectively hold promise for addressing GBM’s heterogeneity and improving treatment outcomes.

### 4.2 BBB

The BBB remains one of the most formidable obstacles in GBM treatment, limiting the delivery of therapeutic agents to the brain. Although advances in polymeric NP engineering have improved BBB penetration, achieving sufficient drug concentrations at the tumor site remains a challenge.

Overcoming the BBB remains a significant challenge in GBM treatment. Nanotechnologies such as convection-enhanced delivery (CED), focused ultrasound (FUS), and intranasal delivery have shown promise in addressing this barrier (Lim et al., 2024). CED infuses drugs directly into brain tissue under convection pressure, enhancing local drug concentration and reducing systemic toxicity. Studies indicate that combining CED with NPs enhances drug distribution and efficacy in GBM (Lim et al., 2023). FUS temporarily opens the BBB by focusing ultrasound energy, which increases drug permeability in brain tissue; for instance, combining FUS with drug-loaded NPs raises local drug concentration (Liu et al., 2022b). Intranasal delivery, a non-invasive method that transports drugs to the brain via the nasal cavity, bypasses the BBB to achieve rapid, efficient drug delivery. Studies suggest that intranasal delivery of NPs enhances drug distribution and therapeutic effects in brain tissue (Maher et al., 2023). In addition, future strategies focus on engineering polymeric NPs with enhanced BBB-crossing capabilities, such as ligand-functionalized NPs targeting receptors overexpressed on endothelial cells, including TfR and LDLR (Kang et al., 2018). Bioinspired approaches, such as coating NPs with cell membranes (e.g., red blood cells or macrophages), improve biocompatibility and leverage natural transcytosis mechanisms to cross the BBB efficiently. Additionally, personalized delivery systems tailored to patient-specific BBB integrity and tumor profiles are under development, aiming to optimize therapeutic efficacy. Advanced imaging and theranostic capabilities integrated into NPs also allow real-time monitoring of BBB crossing and drug distribution, accelerating the translation of these nanotechnologies into clinical practice (Tang et al., 2019). These innovations are critical for overcoming BBB-associated challenges in GBM treatment.

### 4.3 TME

The TME of GBM is highly complex and dynamic, presenting significant challenges for polymer-based nanotechnology. The TME is characterized by hypoxia, acidic pH, dense extracellular matrix (ECM), and immunosuppressive components such as Tregs and M2-polarized macrophages. Hypoxia limits the effectiveness of therapies reliant on ROS generation, such as photodynamic therapy or radiation sensitizers delivered via polymeric NPs. Additionally, the acidic pH of the TME can destabilize some NP formulations, leading to premature drug release. The ECM creates a physical barrier, hindering the penetration and distribution of polymeric NPs throughout the tumor. Furthermore, the immunosuppressive environment reduces the efficacy of immune-modulating agents delivered by NPs, as it suppresses anti-tumor immune responses. Variability in the TME across different patients and tumor regions adds another layer of complexity, making it challenging to design universally effective NP systems (Wolf et al., 2019). These obstacles necessitate innovative approaches to optimize NP performance within the TME.

In order to address the challenges posed by the TME, innovative strategies have been explored. The development of stimuli-responsive polymeric NPs capable of adapting to TME conditions, such as hypoxia or acidic pH, is a promising direction. For example, hypoxia-responsive NPs can release therapeutic agents only in oxygen-deprived regions, enhancing localized treatment while minimizing systemic side effects (Kumari et al., 2020). To overcome the physical barrier of the ECM, NPs can be functionalized with ECM-degrading enzymes, such as hyaluronidase, to improve tumor penetration (Mohiuddin and Wakimoto, 2021). For immunosuppressive TMEs, polymeric NPs can deliver immune checkpoint inhibitors or cytokines to reprogram immune cells, shifting macrophages from the M2 phenotype to the pro-inflammatory M1 phenotype and enhancing T-cell activation (Liu et al., 2022a). Additionally, bioinspired approaches, such as coating NPs with tumor-derived vesicles, can enable better navigation through the TME.

### 4.4 Biocompatibility and safety

Biocompatibility and safety remain critical challenges in the application of polymer-based nanotechnology for GBM treatment (Mu et al., 2024). Although many polymeric NPs are designed using biocompatible materials, such as PEG or PLGA, their degradation products can still pose risks, including inflammation or toxicity in surrounding tissues (Ozdemir-Kaynak et al., 2018). Immunogenicity is another concern, as some surface modifications, such as PEGylation, may induce hypersensitivity reactions or accelerate immune clearance upon repeated administration. Additionally, the long-term accumulation of polymeric NPs in the brain or other organs, such as the liver or spleen, may lead to off-target toxicity or unforeseen side effects. The release of therapeutic payloads in an uncontrolled manner, such as premature drug release, can exacerbate systemic toxicity while reducing efficacy. These challenges highlight the need for careful material selection, improved surface engineering, and comprehensive preclinical safety evaluations before clinical translation.

To address the challenges of biocompatibility and safety in polymer-based nanotechnology for GBM treatment, future advancements focus on designing safer and more efficient NPs (Lim et al., 2024; Khan et al., 2022). The use of fully biodegradable and FDA-approved polymers, such as PLGA and polycaprolactone, can minimize toxicity concerns (Jena et al., 2020). Incorporating bioinspired materials, such as cell membrane coatings or extracellular vesicle-like NPs, can enhance biocompatibility and reduce immune responses (Wang et al., 2023b). Advanced surface engineering strategies, such as zwitterionic coatings, can improve NP stability while minimizing hypersensitivity reactions and immune clearance. Stimuli-responsive NPs capable of precise, localized drug release can mitigate systemic toxicity by ensuring therapeutic agents are delivered only within the TME. Comprehensive preclinical studies using advanced models, such as organoids or patient-derived xenografts, will help predict long-term safety profiles. Regulatory frameworks must also adapt to accommodate the unique properties of nanomedicines, ensuring robust and consistent safety evaluations for clinical applications. These strategies aim to ensure that polymeric NPs are both effective and safe for GBM patients.

In conclusion, addressing these challenges requires multidisciplinary approaches and collaborative efforts between material scientists, biologists, and clinicians. Other innovative solutions, such as personalized NP systems tailored to patient-specific tumor profiles, advanced manufacturing techniques for scalable production, and improved preclinical models for evaluation, are essential for overcoming these barriers (Liu et al., 2022b). Successfully addressing these issues will pave the way for translating polymer-based nanotechnology into effective and accessible treatments for GBM.

## 5 Conclusion

Polymer-based nanotechnology has emerged as a transformative approach in GBM treatment, addressing critical challenges associated with conventional therapies. By leveraging the unique properties of polymeric NPs, such as biocompatibility, tunable drug release, and multifunctionality, this technology enables precise and targeted delivery of therapeutic agents. Polymeric NPs have demonstrated potential across diverse applications, including the delivery of chemotherapeutic drugs, targeted drugs, immunotherapeutic agents, and other therapeutic agents like radiosensitizers. Advanced designs, such as stimuli-responsive systems and bioinspired coatings, further enhance the ability of polymeric NPs to overcome the BBB and adapt to the TME.

Despite these advancements, challenges remain. The heterogeneity of GBM, the complexity of the BBB, and the immunosuppressive TME continue to hinder treatment efficacy. Future research should prioritize personalized nanomedicine, innovative targeting strategies, and combination therapies to maximize therapeutic potential. Interdisciplinary collaborations will be essential to accelerate the development of clinically viable polymer-based NPs. In conclusion, polymer-based nanotechnology holds significant promise for revolutionizing GBM treatment. By addressing current challenges and embracing future innovations, this approach offers a path toward more effective and patient-specific therapies for one of the most aggressive and treatment-resistant cancers.
